# Synthetic Studies toward 5,6,7,3′,4′-Monomethoxytetrahydroxyflavones: Synthesis of Pedalitin

**DOI:** 10.3390/molecules29020513

**Published:** 2024-01-19

**Authors:** Koteswara Rao Kamma, Joungmo Cho, Hyo Jun Won, So-Yeon Nam, Ngan Hong Le, Je Hyeong Jung, Kee-In Lee

**Affiliations:** 1 Research and Development Center, Molecules & Materials Co., Ltd., Daejeon 34013, Republic of Korea; 2 Green Chemistry Division, Korea Research Institute of Chemical Technology, Daejeon 34114, Republic of Korea; jmcho@krict.re.kr (J.C.); hnganle@krict.re.kr (N.H.L.); 3 Smart Farm Research Center, Korea Institute of Science and Technology, Gangneung 25451, Republic of Koreajhjung@kist.re.kr (J.H.J.)

**Keywords:** 5,6,7,3′,4′-monomethoxytetrahydroxyflavone, pedalitin, 2′-hydroxychalcone, 3-hydroxyflavone, aerobic oxidation, demethylation, X-ray crystal structure

## Abstract

During the synthetic studies toward 5,6,7,3′,4′-monomethoxytetrahydroxyflavones, a concise pedalitin synthesis procedure was achieved. As previously reported, 6-hydroxy-2,3,4-trimethoxyacetophenone was prepared by Friedel–Crafts acylation of 1,4-dihydroxy-2,6-dimethoxybenzene with boron trifluoride diethyl etherate in acetic acid. When aldol condensation of 6-hydroxy-2,3,4-trimethoxyacetophenone **2b** with vanillin was performed in basic conditions, it produced 2′-hydroxychalcone **3b**, and, surprisingly, along with 3-hydroxyflavone **4** in a considerable amount. We propose that this oxidative cyclization is presumably due to the contribution of a quinone methide, likely to be subjected to aerobic oxidation. The chalcone was then subjected to oxidative cyclization with iodine in dimethyl sulfoxide to afford flavone **5** in good yield. To our delight, serial demethylation of the three methoxy groups at the 5-, 6-, and 3′-positions of **5** proceeded smoothly to produce pedalitin **1**, under hydrogen bromide solution (30% in acetic acid). The crystal structures of 3-hydroxyflavone **4** and pedalitin tetraacetate **6** were unambiguously determined by X-ray crystallography.

## 1. Introduction

Flavonoids possessing the carbon skeleton of flavan represent a large subgroup of the phenolic class of plant-specialized metabolites widely distributed throughout the plant kingdom, and are thus commonly consumed in the diets of humans. These phenolic substances are isolated from a wide range of fruits and vegetables, and thus have extensive structural diversity. Depending on the oxidation status and saturation of the flavan ring, flavonoids are divided into several groups, which include flavones, isoflavones, flavonols, and flavanones [1]. Flavones comprise one of the important subgroup of flavonoids and are very important specialized metabolites involved in plant signaling and defense. There has been increasing interest in the research of flavones recently; they are directly associated with key ingredients of the human diet with significant health benefits, besides their physiological, biochemical, and ecological functions to plants [2]. Furthermore, flavones exhibit diverse potential aids for numerous pathological conditions with antioxidant, antiproliferative, antitumor, and antimicrobial properties. Indeed, flavones have served as a privileged scaffold, with potential applications in medicinal chemistry and drug discovery for multitargeting in complex diseases like cancer, inflammation, cardiovascular disease, diabetes, and various neurodegenerative disorders [3].

Flavones are widely present in leaves, flowers, and fruits as glucosides. In particular, citrus fruits are rich sources of polymethoxyflavones and hydroxylated polymethoxyflavones [4]. Recently, polymethoxyflavones have received growing attention due to their multiple bioactivities, such as metabolic regulatory, immunoregulatory, neuroprotective, and skin protective effects. They are characterized by bearing several methoxy groups attached to their flavone structure and are exclusively found in citrus fruits. Among them, tangeretin, nobiletin, and sinensetin are representative examples. The demethylation of such polymethoxyflavones by human gut bacteria creates a plethora of demethylated metabolites that possess different biological activities [5]. Also, many 5-demethylated polymethoxyflavones have been identified during the drying processes of citrus peels [6].

**Figure 1:**
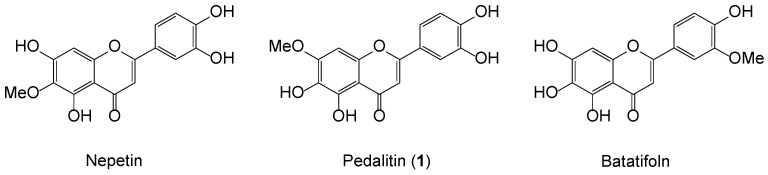
Representative structures of 5,6,7,3′,4′-monomethoxytetrahydroxyflavones.

We recently gave attention to the biological modulations of sinensetin, which possesses strong anti-vascular and anti-inflammatory properties. Sinensetin, 5,6,7,3′,4′-pentamethoxyflavone was identified as, interestingly, leading a unique family of the monomethoxytetrahydroxyflavones comprising nepetin (5,7,3′,4′-OH, 6-OMe), pedalitin (7-OMe), and batatifolin (3′-OMe), as shown in Figure 1 [7]. Most flavonoid synthesis is heavily dependent on methoxylated acetophenones and benzaldehydes, as readily available aldol partners. However, the selective demethylation of polymethoxyflavones is hard to achieve and thus randomly selected from the vast pool of Brønsted and Lewis acids, depending upon the location and number of methoxy groups on the flavone ring [8,9,10]. Due to the lack of selective demethylation, a protection–deprotection strategy is frequently required to avoid such complexity [11].

**Figure 2:**
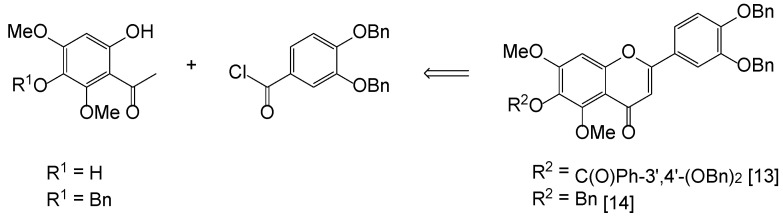
Previous synthesis of pedalitin.

Pedalitin (**1**) drew our consideration as the first synthetic target for chemical modifications, since it is an inhibitor of tyrosinase (IC_50_ = 0.28 mM) and α-glucosidase (IC_50_ = 0.29 mM) [12]. Surprisingly, only a few syntheses of pedalitin have been disclosed, and two accessible reports are summarized in Figure 2. These approaches are mainly dependent upon protection and deprotection methods through Baker–Venkataraman rearrangement [13,14]. Furthermore, the preparation of two intermediates, 3-benzyloxy-6-hydroxy-2,4-dimethoxyacetophenone (R^1^ = Bn) and 3,4-bis(benzyloxy)benzoyl chloride, requires multiple transformations. The initial acetophenone (R^1^ = H) was obtained from 2,6-dimethoxy-1,4-benzoquinone after reductive acetylation using zinc powder and acetic anhydride followed by a Fries rearrangement. Additionally, selective benzylation of the initial gave 3-benzyloxy-6-hydroxy-2,4-dimethoxyacetophenone [15]. The second partner was prepared from commercially available 3,4-dihydroxybenzoic acid, which was per-benzylated and the resulting ester hydrolyzed, and then the acid was converted to 3,4-bis(benzyloxy)benzoyl chloride [16].

Here, we would like to report the concise synthesis of pedalitin starting from readily available and cheap materials, 3,4,5-trimethoxyphenol and vanillin. During aldol condensation of 6-hydroxy-2,3,4-trimethoxyacetophenone and vanillin, we found an unusual formation of 3-hydroxyflavone along with 2′-hydroxychalcone in the basic conditions. We propose that this oxidative cyclization is presumably due to the contribution of a quinone methide, likely to be subjected to aerobic oxidation. The 2′-hydroxychalcone was then subjected to oxidative cyclization to give 4′-demethylated sinensetin. At the final installation, a series of demethylation processes of the flavone was successfully carried out to produce pedalitin, when employing hydrogen bromide. The crystal structures of 3-hydroxyflavone and pedalitin (as its tetraacetate) were unambiguously determined.

## 2. Results and Discussion

**Figure 3:**
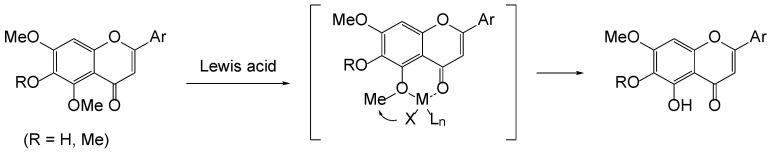
Lewis acid-mediated 5-demethylation of flavonoid.

We initially anticipated that the demethylation of 5-methoxyflavon could be controlled by the neighboring group participation of the 4-carbonyl group, as demonstrated in Figure 3. Indeed, 5-demethylflavones have been commonly produced employing Lewis acids such as AlCl_3_ [17,18], MgBr_2_.OEt_2_ [19], and BCl_3_ [20]. Similarly, such demethylation was observed with a Brønsted acid [21]. We next postulate that demethylation takes place firstly at the 5-methoxy by the presence of the 4-carbonyl and then takes over the next nearest methoxy group (if, R = Me) by a newly formed 5-hydroxy group in both Brønsted and Lewis acids. It has been revealed that the 7-methoxy of 5,6,7-trimethoxyflavones is much harder to remove than the 5- and 6-methoxy groups [22,23].

**Scheme 1:**
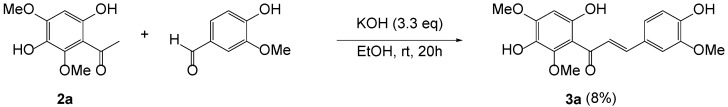
Aldol condensation of **2a** with vanillin.

As previously reported, 3,6-dihydroxy-2,4-dimethoxyacetophenone **2a** was prepared by Friedel–Crafts acylation of 1,4-dihydroxy-2,6-dimethoxybenzene with boron trifluoride diethyl etherate in AcOH [24]. When aldol condensation of **2a** with vanillin was carried out in the presence of KOH in the protic conditions (Scheme 1), it produced an extremely low yield of **3a** as previously reported [25]. The use of several different kinds of bases, such as pyrrolidine, NaOH, NaH, KOBu*^t^*, and LiHMDS, did not improve yield at all.

We next prepared 6-hydroxy-2,3,4-trimethoxyacetophenone **2b** in a 76% yield by the Friedel–Crafts acylation of 3,4,5-trimethoxyphenol, as reported earlier [24]. When aldol condensation of **2b** with vanillin was performed in the basic medium, it produced aldol **3b** and surprisingly a cyclized product **4** in a considerable amount (entries 1 and 2). The latter’s structure was determined by X-ray crystallography, which was proven to be 3-hydroxyflavone, as shown in Table 1. We next examined several bases in an aprotic solvent like THF; however, every case resulted in lesser amounts of **4** (entries 3–5). We could avoid the formation of **4** as strict freeze–pump–thaw degassing was applied under an inert atmosphere (entry 6). These results suggest that molecular oxygen is possibly involved in this transformation.

The formation of flavonol is quite unusual compared to 4-methoxylated vanillin analogs as the aldehyde partners [26,27]. It should be mentioned that flavonols have been commonly prepared from 2′-hydroxychalcones through oxidative cyclization mediated by hydrogen peroxide in an alkaline medium, referred to as the Algar–Flynn–Oyamada reaction [28]. Interestingly enough, a synthesis of flavonols from 2′-hydroxyacetophenones and benzaldehydes promoted by pyrrolidine under an aerobic condition in water has recently appeared [29].

**Scheme 2:**
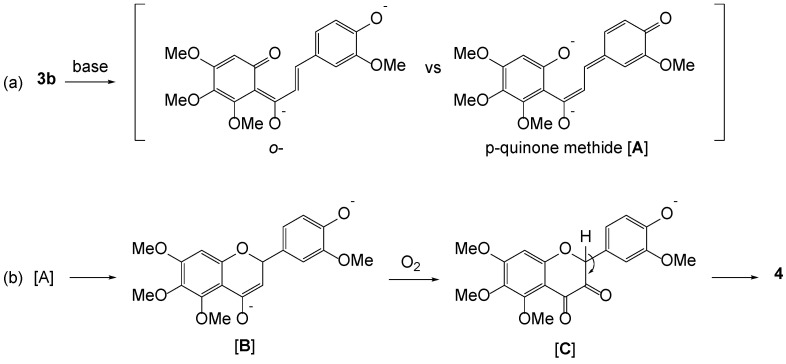
(**a**) *o*- versus *p*-quinone methide. (**b**) A plausible mechanism for aerobic oxidation of **3**.

We thus propose that this unusual oxidative cyclization is presumably due to the contribution of a quinone methide species as illustrated in Scheme 2. Under the basic conditions, two different kinds of quinone methides from **3b** are possible. However, we assumed that *p*-quinone methide **A** is more likely to be subjected to aerobic oxidation than that of *o*-quinone methide since 4-methoxylated vanillin analogs solely produce the aldol products. *p*-Quinone methide could be prone to intramolecularly undergo ‘ring closure’ to **B** by the attack of phenoxide in a 1,6-conjugated addition [30]. Seemingly, the enolate is labile to take up air oxidation affording 1,2-diketone species **C** via a peroxide intermediate, which is readily enolizable to 3-hydroxylflavon **4** [29,31]. However, more precise understandings of this transformation are required.

On the other hand, the intramolecular cyclization of 2’-hydroxychalcone **3,** mediated by I_2_ in DMSO, smoothly yielded **5** in a 74% yield. For a series of demethylation of **5**, however, initial approaches employing Lewis acid-mediated demethylation ended up with complex mixtures, when it allowed for a prolonged time. We next examined HBr (30% in AcOH) as the demethylating agent. Astonishingly, serial demethylation of three methoxy groups of **5** proceeded accordingly to produce a flavone holding one methoxy in a 45% yield.

**Scheme 3:**
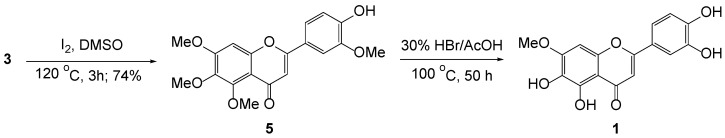
Synthesis of pedalitin **1**.

During the demethylation of **5** with HBr in AcOH, we carefully monitored the progress of the reaction by TLC at time intervals. A crude aliquot was regularly withdrawn from the reaction media and recorded by ^1^H NMR and LC-MS. At the initial stage, the mixture showed the consumption of the starting material and multiple spots due to random demethylation of the existing methoxy groups. After 20 h, the progress showed the consumption of multiple spots and enriched a major spot. A crude sample showed distinctively two methoxy groups in NMR; however, the structure was not firmly determined. No further change was observed by 12 h. After the addition of more agents, the reaction slowly proceeded and reached the final stage, showing one methoxy group after an additional 30 h. It required 50 h around 100 °C for the completion (Scheme 3). We thus observed that sequential demethylation proceeded in quite a time-dependent manner.

However, it was very hard to identify the structure simply compared to the physical and spectroscopic data previously reported, since several monomethoxyflavones are possible. Attempted approaches to produce a single crystal directly the product or its cocrystals with coformers [32,33] such as isoniazid, isonicotinamide, and caffeine were fruitless. The problem was solved, to our delight, as its tetraacetate was suitable for X-ray analysis.

**Figure 4:**
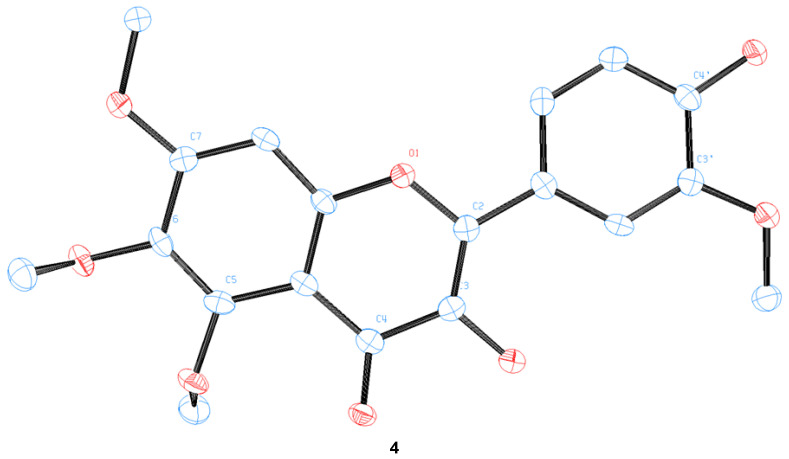

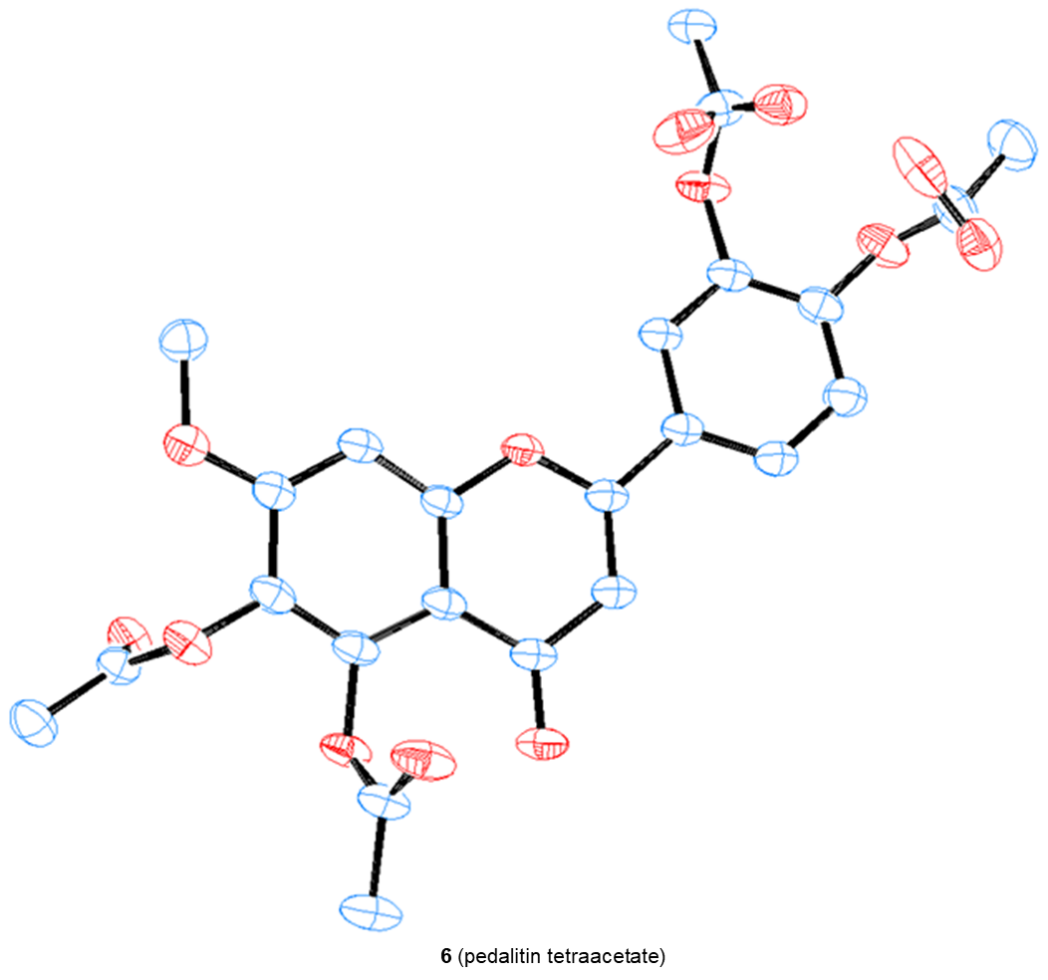
The crystal structures of 3-hydroxyflavone **4** and pedalitin tetraacetate **6**.

We were able to obtain two single crystals suitable for X-ray analysis and unambiguously determine the structure of **4** and pedalitin (as tetraacetate, **6**), as depicted in Figure 4. 3-Hydroxylflavon **4** was purified by recrystallization, and its single crystal was grown by slow evaporation in the MeOH and EtOH mixture. To determine the structure, pedalitin was fully acetylated under the standard conditions to afford its tetraacetate **6**, and then a single crystal was also similarly obtained by slow evaporation in EtOH. The structure of **4** was proven to be 3-hydroxyflavone showing two intramolecular hydrogen bonds between 3-OH and 4-C(O), and 3′-OMe and 4′-OH. The tetraacetate clearly shows the structure of pedalitin. However, the two carbonyl oxygens of 3′- and 4′-acetate of the tetraacetate show disorder. Interestingly, the dihedral angle of 3(C)-2(C)-1′(C)-2′(C) takes near-to-flat geometry as −11.6(6) for **4** and 166.9(18) for the tetraacetate compared to those of 2-phenylphenol [34] and biphenyl [35], respectively. It should be mentioned that there are many instances of a series of demethylations of polymethoxyflavones with widely contrasting outcomes [22,23,36,37].

## 3. Materials and Methods

### 3.1. General

All solvents and reagents were purchased from commercial sources and used as received without further purification unless otherwise stated. Potassium phosphate was crushed in a mortar and dried at 70 °C in an oven overnight and used. Reactions were monitored by thin-layer chromatography carried out on S-2 0.25 mm E. Merck silica gel plates (60F-254) using UV light as the visualizing agent and an acidic mixture of anisaldehyde or a ninhydrin solution in ethanol and heat as developing agents. E. Merck silica gel (60, particle size 0.040–0.063 mm) was used for flash column chromatography. All yields were calculated from isolated products.

All NMR spectra were recorded on Bruker AV-500 instrument (Bruker Scientific LLC, Billerica, MA, USA). ^1^H and ^13^C NMR spectra were referenced internally to the residual undeuterated chloroform (δH = 7.26 ppm and δC = 77.3 ppm) and dimethyl sulfoxide (δH = 2.54 ppm and δC = 40.4 ppm). The NMR data were analyzed using MNova 10.0 processing software (Mestrelab Research, San Diego, CA, USA). The following abbreviations were used to designate multiplicities: s = singlet, d = doublet, t = triplet, q = quartet, quint = quintet, m = multiplet, and br s = broad singlet. Chemical shifts are reported in ppm and coupling constants are in Hertz (Hz).

Low-resolution mass spectra were obtained using liquid chromatography–mass spectrometer (LCMS) with a Waters ACQUITY UPLC H-Class/SQD2 Mass Spectrometer (Waters Corporation, Milford, MA, USA). High-resolution mass spectra (HRMS) were obtained using LC-TOF mass spectrometer on Waters LCT Premier XE Mass Spec system (Waters Corporation, Milford, MA, USA) with electrospray ionization (ESI) in positive or negative mode depending on the analytes. HRMS data analysis was performed with MassLynx Applications.

The data for X-ray structure determination were collected on Bruker SMART Apex II X-ray diffractometer instrument (Bruker Scientific LLC, Billerica, MA, USA) equipped with graphite-monochromated MoKα radiation (λ = 0.71073 Å). The data were collected at the low temperature of 100 K by the *ϕ*–*ω* scan method. The collected data were integrated by using Bruker SAINT software (V7.06A) and an absorption correction was not applied. The structure was solved and refined through the least-squares method with SHELXT and SHELXL programs, respectively. All the non-hydrogen atoms were refined anisotropically and hydrogen atoms were placed in calculated positions.

### 3.2. Preparation of 3,6-Dihydroxy-2,4-dimethoxyacetophenone ***2a*** and 6-hydroxy-2,3,4-trimethoxyacetophenone ***2b***

The starting materials, **2a** and **2b,** were prepared by Friedel–Crafts acylation of 1,4-dihydroxy-2,6-dimethoxybenzene and 3,4,5-trimethoxyphenol, respectively, in acetic acid with boron trifluoride diethyl etherate as previously documented [24].

**2a**: 1.75 g (pale yellow solid, 71.2% yield); ^1^H NMR (500 MHz, CDCl_3_) δ 13.21 (s, 1H), 6.29 (s, 1H), 5.12 (s, 1H), 3.98 (s, 3H), 3.95 (s, 3H), 2.70 (s, 3H); LCMS (ESI+): *m*/*z* = 213.1 [M + H]^+^.

**2b**: 2.73 g (pale yellow solid, 75.7% yield); ^1^H NMR (500 MHz, CDCl_3_) δ 13.45 (s, 1H), 6.24 (s, 1H), 4.00 (s, 3H), 3.89 (s, 3H), 3.79 (s, 3H), 2.66 (s, 3H); ^13^C NMR (125 MHz, CDCl_3_) δ 203.3, 161.8, 160.0, 155.2, 134.7, 108.4, 96.0, 60.9, 60.9, 56.0, 31.8; LCMS (ESI+): *m*/*z* = 227.2 [M + H]^+^.

### 3.3. Aldol Condensation of ***2a*** with Vanillin (Scheme 1)

To a solution of **2a** (940 mg, 4.42 mmol) in EtOH (5 mL) vanillin (820 mg, 5.38 mmol) and powdered KOH (820 mg, 14.6 mmol) were added, and the mixture was stirred at room temperature for 20 h. The reaction mixture was adjusted to a pH value of ~4 using 1N HCl, and then extracted with EtOAc (30 mL × 2). The combined organic layer was dried over anhydrous Na_2_SO_4_, filtered, and concentrated in vacuo. The crude was subjected to column chromatography (5–40% EtOAc in hexanes) to afford 125 mg of **3a** (8.2% yield) as an off-white solid.

**3a**: ^1^H NMR (500 MHz, CDCl_3_) δ 13.39 (s, 1H), 7.84 (s, 2H), 7.21 (dd, *J* = 15.2, 1.5 Hz, 1H), 7.16 (d, *J* = 2.3 Hz, 1H), 6.98 (d, *J* = 15.2 Hz, 1H), 6.35 (s, 1H), 5.97 (s, 1H), 5.26 (s, 1H), 3.97 (s, 3H), 3.96 (s, 3H), 3.88 (s, 3H); LCMS (ESI+): *m*/*z* = 347.2 [M + H]^+^.

### 3.4. Aldol Condensation of ***2b*** with Vanillin (Entry 2, Table 1)

To a solution of **2b** (1.0 g, 4.42 mmol) in EtOH (6 mL) was added vanillin (810 mg, 5.32 mmol) and powdered KOH (740 mg, 13.1 mmol), and the mixture was heated to reflux for 20 h. The reaction mixture was cooled to room temperature, adjusted to a pH value of ~4 using 1N HCl, and then extracted with EtOAc (30 mL × 2). The combined organic layer was dried over anhydrous Na_2_SO_4_, filtered, and concentrated in vacuo. The crude was subjected to column chromatography (5–30% EtOAc in hexanes) to afford 450 mg of **3b** (off-white solid, 28.3%) and 340 mg of **4** (yellow solid, 20.5% yield).

**3b**: ^1^H NMR (500 MHz, DMSO-*d*_6_) δ 12.23 (s, 1H), 9.77 (brs, 1H), 7.55 (d, *J* = 15.0 Hz, 1H), 7.40 (d, *J* = 15.0 Hz, 1H), 7.28 (s, 1H), 7.18 (d, *J* = 10.0 Hz, 1H), 6.83 (d, *J* = 10.0 Hz, 1H), 6.38 (s, 2H), 3.84 (s, 6H), 3.83 (s, 3H), 3.71 (s, 3H); ^13^C NMR (125 MHz, DMSO-*d*_6_) δ 192.9, 158.3, 158.2, 153.5, 150.1, 148.4, 144.9, 135.1, 126.6, 124.3, 123.5, 116.3, 112.2, 110.9, 96.9, 61.9, 61.1, 56.4, 56.1; LCMS (ESI+): *m*/*z* = 361.4 [M + H]^+^.

**4**: ^1^H NMR (500 MHz, DMSO-*d*_6_) δ 9.67 (s, 1H), 9.04 (s, 1H), 7.78 (d, *J* = 1.95 Hz, 1H), 7.73 (dd, *J* = 8.45 Hz, 2.0 Hz 1H), 7.20 (s, 1H), 6.95 (d, *J* = 8.4 Hz, 1H), 3.96 (s, 3H), 3.87 (s, 3H), 3.85 (s, 3H), 3.78 (s, 3H); ^13^C NMR (125 MHz, DMSO-*d*_6_) δ 171.3, 157.9, 153.4, 151.4, 148.8, 147.9, 143.4, 139.7, 137.8, 122.6, 121.6, 116.0, 111.9, 110.3, 97.4, 62.3, 61.5, 56.9, 56.3; LCMS (ESI+): *m*/*z* = 375.3 [M + H]^+^.

### 3.5. Preparation of a Single Crystal of ***4***

Slow evaporation of **4** (10 mg) in MeOH/EtOH (1/1, 6 mL) afforded a single crystal suitable for X-ray analysis.

### 3.6. Intramolecular Cyclization of ***3b*** to ***5***

To a solution of **3b** (150 mg, 0.41 mmol) in DMSO (2 mL), was added I_2_ (10.5 mg, 0.04 mmol), and maintained the reaction mass at 120 °C for 5 h. After completion of the reaction, the mixture was quenched with aqueous NaHSO_3_ and then extracted with EtOAc (20 mL × 2). The combined organic layer was washed with brine, dried over anhydrous Na_2_SO_4_, filtered, and then concentrated to give a residue. The crude was purified by column chromatography (5–20% EtOAc in hexanes) to afford 110 mg of **5** (74.8% yield).

**5**: ^1^H NMR (500 MHz, DMSO-*d*_6_) δ 9.87 (s, 1H), 7.57 (s, 2H), 7.22 (s, 1H), 6.94 (s, 1H), 6.76 (s, 1H), 3.96 (s, 3H), 3.91 (s, 3H), 3.81 (s, 4H), 3.77 (s, 3H); ^13^C NMR (125 MHz, DMSO-*d*_6_) δ 176.1, 161.0, 157.8, 154.3, 152.0, 150.6, 148.4, 140.1, 122.2, 120.2, 116.1, 112.4, 110.3, 106.2, 97.7, 62.3, 61.4, 56.8, 56.4; ESI-HRMS *m*/*z* [M + Na]^+^ calcd for C_19_H_18_NaO_7_ 381.0950; found, 381.0949.

### 3.7. Demethylation of ***5*** to Pedalitin ***1***

A solution of **5** (210 mg, 0.58 mmol) in 30% HBr in acetic acid (2.35 mL, 11.9 mmol) was heated to 120 °C. The progress of the reaction was carefully monitored by TLC at time intervals. A crude aliquot was regularly withdrawn from the reaction media and recorded by ^1^H NMR and/or LC-MS:

(i) The reaction showed the consumption of starting material and multiple spots on TLC after 8 h;
(ii) After 20 h, the reaction showed the consumption of multiple spots and enriched a major spot apparently;
(iii) No further progress was observed after an additional 12 h.

A withdrawn sample was recorded by ^1^H NMR showing distinctively two methoxy groups and three hydroxyl protons. However, we could not judge for specified sites of the two remaining methoxy groups at the current stage; {^1^H NMR (500 MHz, DMSO-d_6_) δ 12.6 (s, 1H), 9.98 (s, 1H), 8.73 (s, 1H), 3.92 (s, 6H); LCMS (ESI+): *m*/*z* = 331.3 [M + H]^+^}.

After the reaction temperature was set at 80 °C, 30% HBr in acetic acid (2.80 mL, 14.2 mmol) was added and then the mixture was allowed to proceed for a long time. Finally, a decent conversion was observed after an additional 30 h. The solution was concentrated in vacuo and the residue was purified by column chromatography twice (10–30% MeOH in dichloromethane) to afford **1** (83 mg, 45.3% yield).

**1**: ^1^H NMR (500 MHz, DMSO-*d*_6_) δ 12.6 (s, 1H), 9.97 (s, 1H), 9.38 (s, 1H), 8.73 (s, 1H), 7.45 (s, 2H), 6.92–6.87 (m, 2H), 6.71 (s, 1H), 3.93 (s, 3H); ^13^C NMR (125 MHz, DMSO-*d*_6_) δ 182.5, 164.4, 154.7, 150.08, 150.06, 146.70, 146.23, 130.8, 122.2, 119.3, 116.4, 113.9, 105.4, 103.0, 91.4, 56.7; ESI-HRMS *m/z* [M + Na]^+^ calcd for C_16_H_12_NaO_7_ 339.0481; found, 339.0479.

### 3.8. Crystal Structure Determination of ***1*** (as Tetraacetate ***6***)

Acetylation of pedalitin was carried out under the standard conditions (Ac_2_O, pyridine, rt, 12 h) to give its tetraacetate **6** in 94.7% yield. Slow evaporation of the tetraacetate (5 mg) in EtOH (2 mL) afforded a single crystal suitable for X-ray analysis.

**6**: ^1^H NMR (500 MHz, CDCl_3_) δ 3.95 (s, 3H), 2.44 (s, 3H), 2.35 (s, 6H), 2.34 (s, 3H), 2.33 (s, 3H); ^13^C NMR (125 MHz, CDCl_3_) δ 176.0, 168.7, 168.0, 167.9, 167.8, 160.3, 156.3, 155.6, 144.6, 142.6, 141.8, 130.7, 129.9, 124.4, 124.2, 121.4, 111.2, 108.6, 98.2, 56.6, 20.8, 20.7, 20.6, 20.2.

### 3.9. Crystallographic Data

Atomic coordinates and crystallographic parameters for **6** (CCDC 2285705, Supplementary Material) and **4** (CCDC 2285707, Supplementary Material) were deposited at the Cambridge Crystallographic Data Center. These data can be obtained free of charge via http://www.ccdc.cam.ac.uk/conts/retrieving.html, or by contacting the CCDC, 12 Union Road, Cambridge CB2 1EZ, UK; Fax: +44 1223 336033; E-mail: deposit@ccdc.cam.ac.uk. The crystallographic data are summarized in Table 2.

## 4. Conclusions

Polymethoxyflavones such as tangeretin, nobiletin, and sinensetin have attracted much attention due to their multiple bioactivities as well as potential health benefits. Moreover, the demethylation of such polymethoxyflavones provides a vast pool of demethylated flavones, which has been suggested to significantly influence the bioactivities. Among them, sinensetin leads a unique family of exclusively only one methoxy group-containing congeners consisting of nepetin, pedalitin, and batatifolin. Interestingly enough, these flavones draw our attention meanwhile they have not been much studied from a synthetic point of view.

In this study, we achieved a concise synthesis of pedalitin beginning from readily available and cheap materials, 3,4,5-trimethoxyphenol and vanillin. The key precursor **5** was conveniently prepared from 6-hydroxy-2,3,4-trimethoxyacetophenone **2b**, sequentially employing aldol condensation with vanillin in the basic conditions and then iodine-catalyzed intermolecular cyclization of the resulting chalcone **3b**. Under 30% HBr solution, serial demethylation of three methoxy groups at 5-, 6-, and 3′-positions of **5** proceeded consecutively to produce pedalitin **1**. We observed that the demethylation proceeds in quite a time-dependent way. The structure was confirmed by X-ray crystallography as its tetraacetate **6**. Conventionally, such flavones bearing monomethoxy and polyhydroxy groups have been assigned compared to physical and spectroscopic data previously reported. However, we feel X-ray crystal structure determination is necessary since they are hardly distinguishable, and some of them should be revised.

Moreover, we found the unusual formation of 3-hydroxyflavone **4** during the aldol condensation along with the chalcone **3b**, and the crystal structure of **4** has been unambiguously determined by X-ray crystallography. We propose that this oxidative cyclization is presumably due to the contribution of a quinone methide, likely to be subjected to aerobic oxidation. Currently, more detailed understandings of aerobic oxidation using model compounds are underway.

## Figures and Tables

**Table 1:** Aldol condensation of **2b** with vanillin.

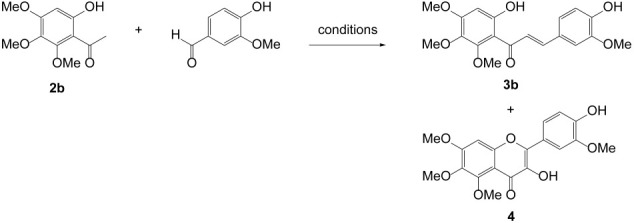		
Entry ^a^	Conditions	Yield (%) of 3b/4
1	KOH (3.3 eq), EtOH/H_2_O (5/2), rt, 20 h	15/10
2	KOH (3.0 eq), EtOH, reflux, 20 h	28/20
3	KOBu*^t^* (3.0 eq), THF, rt, 12 h	12/5
4	NaH (3.0 eq), THF, rt, 12 h	15/8
5	LiHMDS (3.5 eq), THF, −78 °C to rt, 20 h	18/3
6	THF; freeze-pump-thaw (3 cycles); LiHMDS (3.5 eq), −78 °C to rt, 20 h; under Ar	23/-

^a^ Reaction conditions: **2b** (1.0 g, 4.42 mmol), vanillin (1.2 eq).

**Table 2:** Crystallographic data of **4** and **6**.

Geometric Data	4	6
Empirical formula	C_19_H_18_O_8_	C_24_H_20_O_11_
Formula weight	374.33	484.40
Crystal system	Orthorhombic	triclinic
Space group	Pna21	P-1
Unit cell dimensions	a = 8.2312(19) Å	a = 8.94440(10) Å
	b = 16.306(3) Å	b = 10.9710(2) Å
	c = 12.099(3) Å	c = 13.8699(2) Å
Volume	1623.9(6) Å^3^	1164.11(4) Å^3^
Density (calculated)	1.531 g/cm^3^	1.382 g/cm^3^
Reflections collected	22,072	35,274
Final R indices [I > 2sigma(I)]	R1 = 0.0492, wR2 = 0.1038	R1 = 0.0543, wR2 = 0.1661
R indices (all data)	R1 = 0.0552, wR2 = 0.1065	R1 = 0.0630, wR2 = 0.1754

## Data Availability

The data presented in this study are available on request from the corresponding authors.

## Supplementary Materials

The following supporting information can be downloaded at: https://www.mdpi.com/article/10.3390/molecules29020513/s1. Online supplementary information contains ^1^H and ^13^C NMR spectra for all compounds prepared in this study. CCDC 2285705 and 2285707 contain supplementary crystallographic data for this paper.
