# Supplementary material for: Synthetic Studies toward 5,6,7,3′,4′-Monomethoxytetrahydroxyflavones: Synthesis of Pedalitin
Source: Molecules. 2024 Jan 19;29(2):513. doi: 10.3390/molecules29020513 (PMC10821304; doi:10.3390/molecules29020513)
Supplement: Supplementary file 1 [file molecules-29-00513-s001.zip › molecules-2747217-SM.pdf]

Supplementary Material

Synthetic studies toward 5,6,7,3',4'-monomethoxytetrahydroxyflavones: Synthesis of Pedalitin

Koteswara Rao Kamma[1,†], Joungmo Cho[2,†], Hyo Jun Won[3], So-Yeon Nam[1], Ngan Hong Le[2], Je Hyeong Jung[3], and Kee-In Lee[1,2,*]

[1] R&D Center, Molecules & Materials Co., Ltd., Daejeon 34013, Republic of Korea

[2] Green Chemistry Division, Korea Research Institute of Chemical Technology, Daejeon 34114, Republic of Korea

[3] Smart Farm Research Center, Korea Institute of Science and Technology, Gangneung, 25451, Republic of Korea

[†] These authors contributed equally to this work.

[*] Author to whom correspondence should be addressed: kilee@krict.re.kr

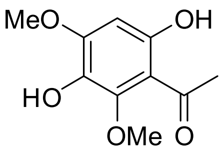
MeO
OH
HO
OMe
O

(**2a**)

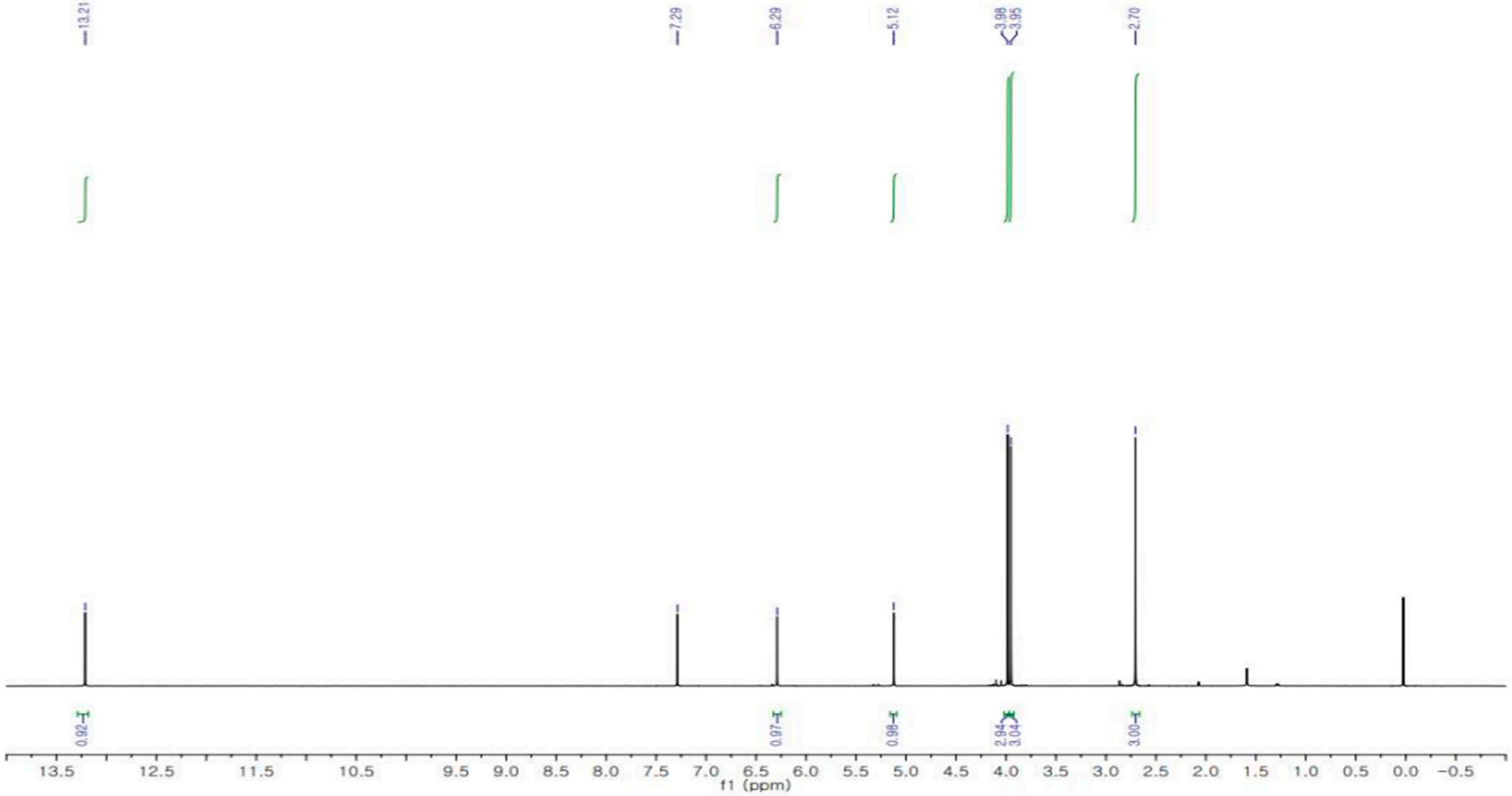
13.21
7.29
6.29
5.12
3.98
3.95
2.70
0.92
0.97
0.98
2.94
3.04
3.00
13.5 12.5 11.5 10.5 9.5 9.0 8.5 8.0 7.5 7.0 6.5 6.0 5.5 5.0 4.5 4.0 3.5 3.0 2.5 2.0 1.5 1.0 0.5 0.0 −0.5
f1 (ppm)

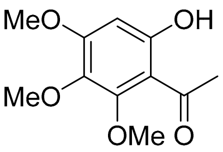
MeO
OH
MeO
OMe O

(**2b**)

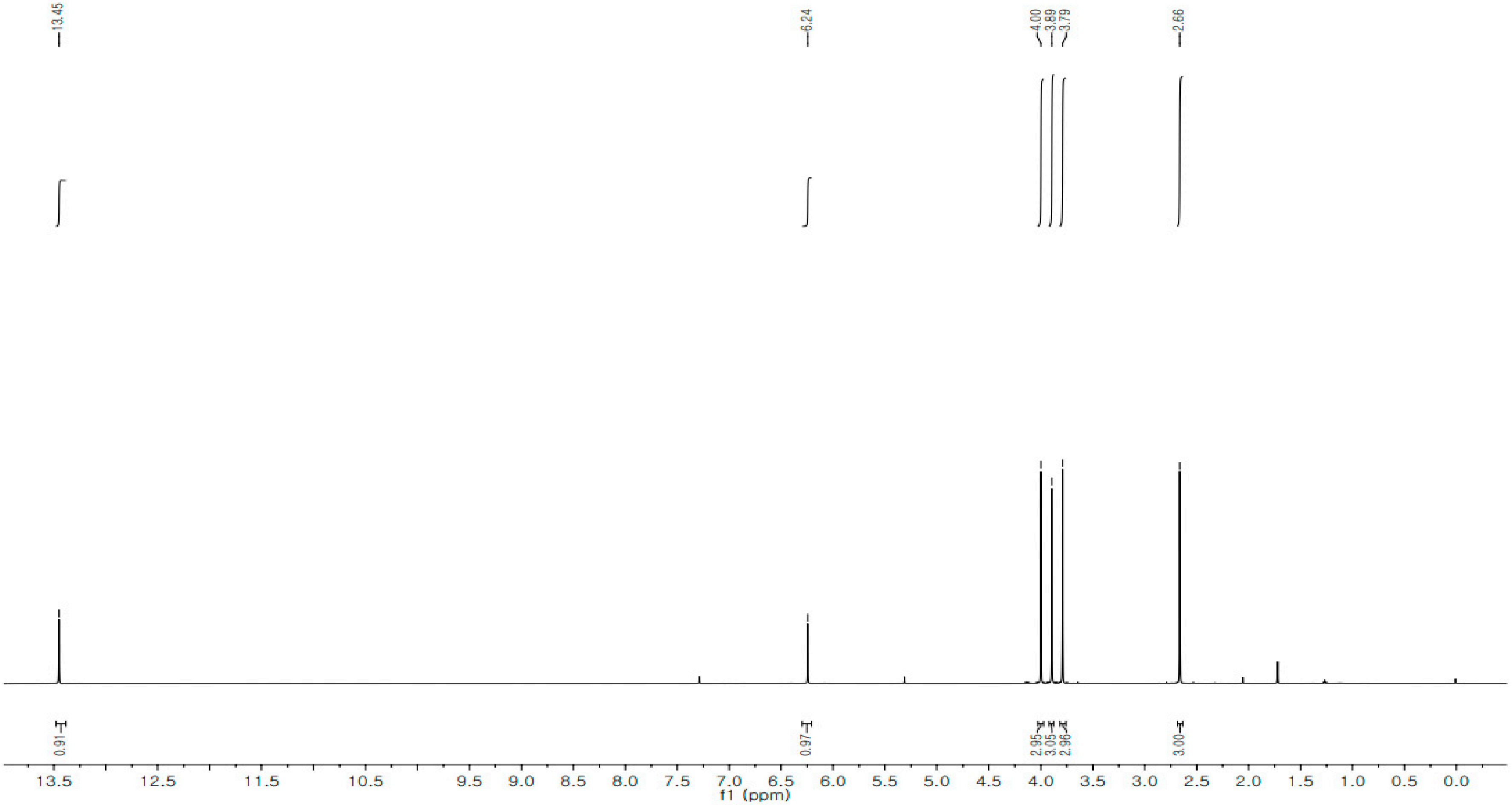
13.45
6.24
4.00
3.89
3.79
2.66
0.91
0.97
2.95
3.05
2.96
3.00
13.5 12.5 11.5 10.5 9.5 9.0 8.5 8.0 7.5 7.0 6.5 6.0 5.5 5.0 4.5 4.0 3.5 3.0 2.5 2.0 1.5 1.0 0.5 0.0
f1 (ppm)

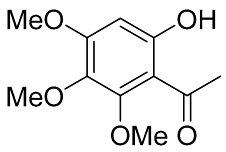

MeO
OH
MeO
OMe
O

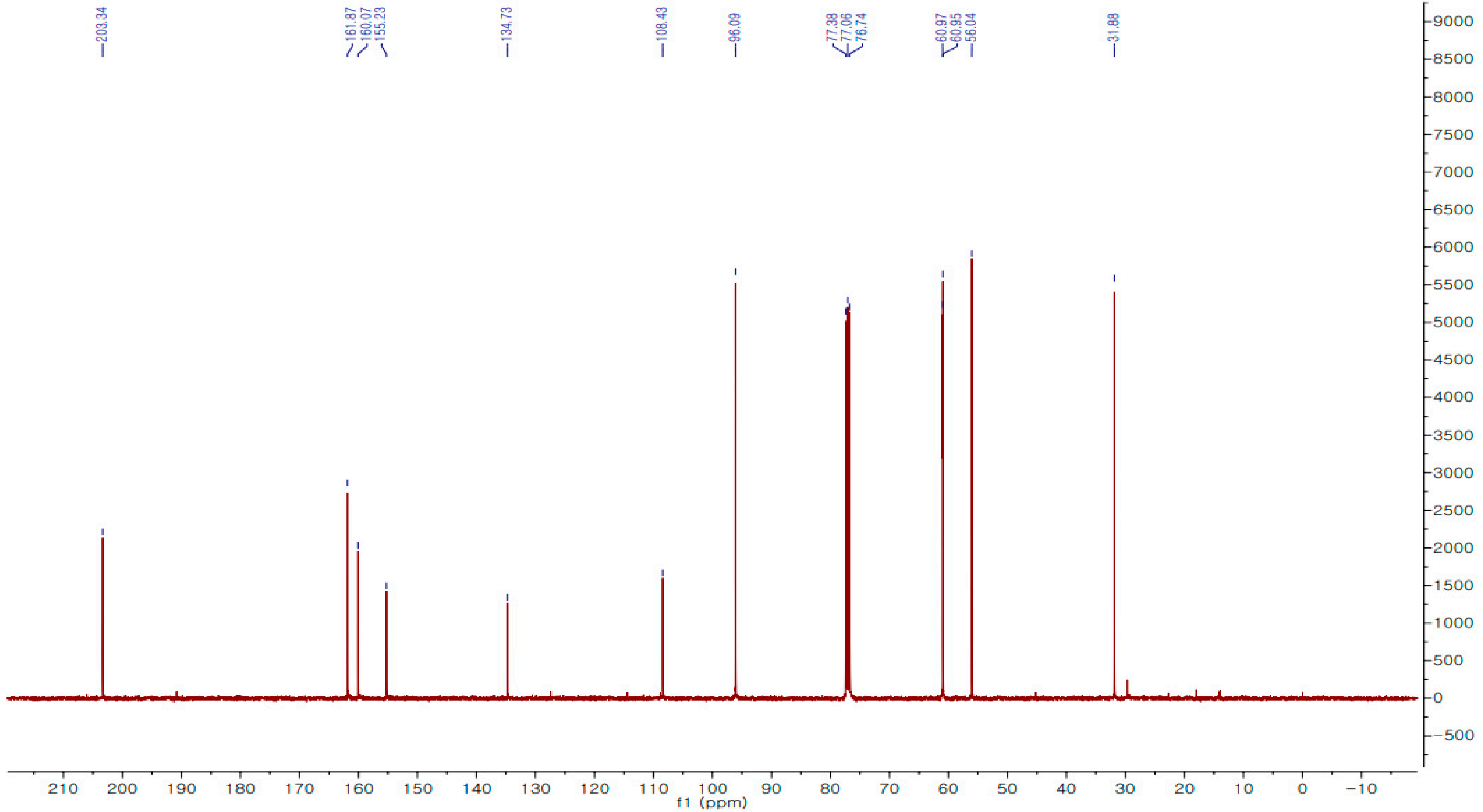

203.34
161.87
160.07
155.23
134.73
108.43
96.09
77.38
77.06
76.74
60.97
60.95
56.04
31.88
f1 (ppm)

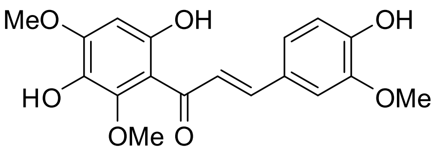
MeO
OH
HO
OMe
O
OH
OMe

(**3a**)

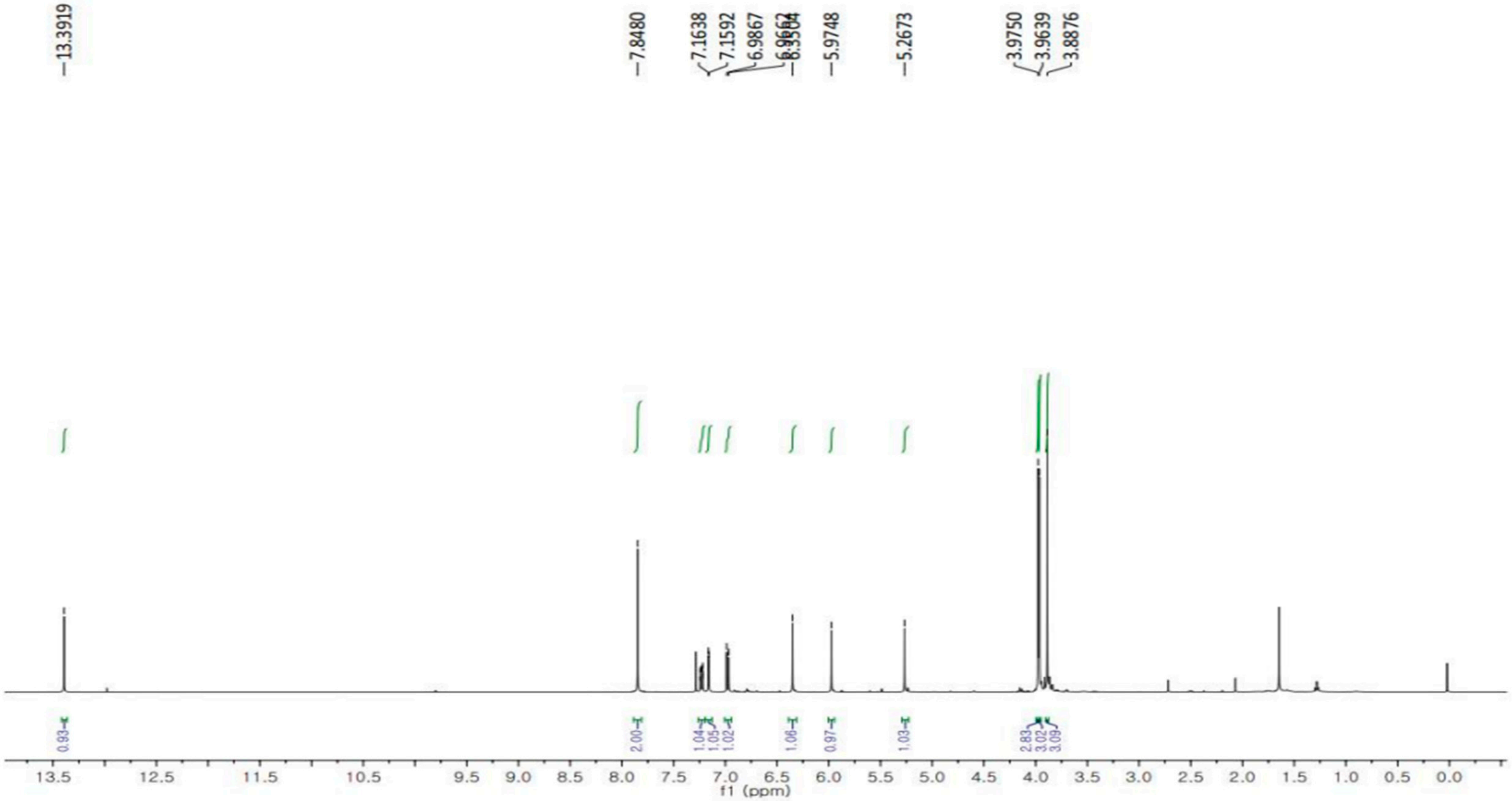
13.3919
7.8480
7.1638
7.1592
6.9867
5.9748
5.2673
3.9750
3.9639
3.8876
0.93
2.00
1.04
1.05
1.02
1.06
0.97
1.03
2.83
3.02
3.09
f1 (ppm)

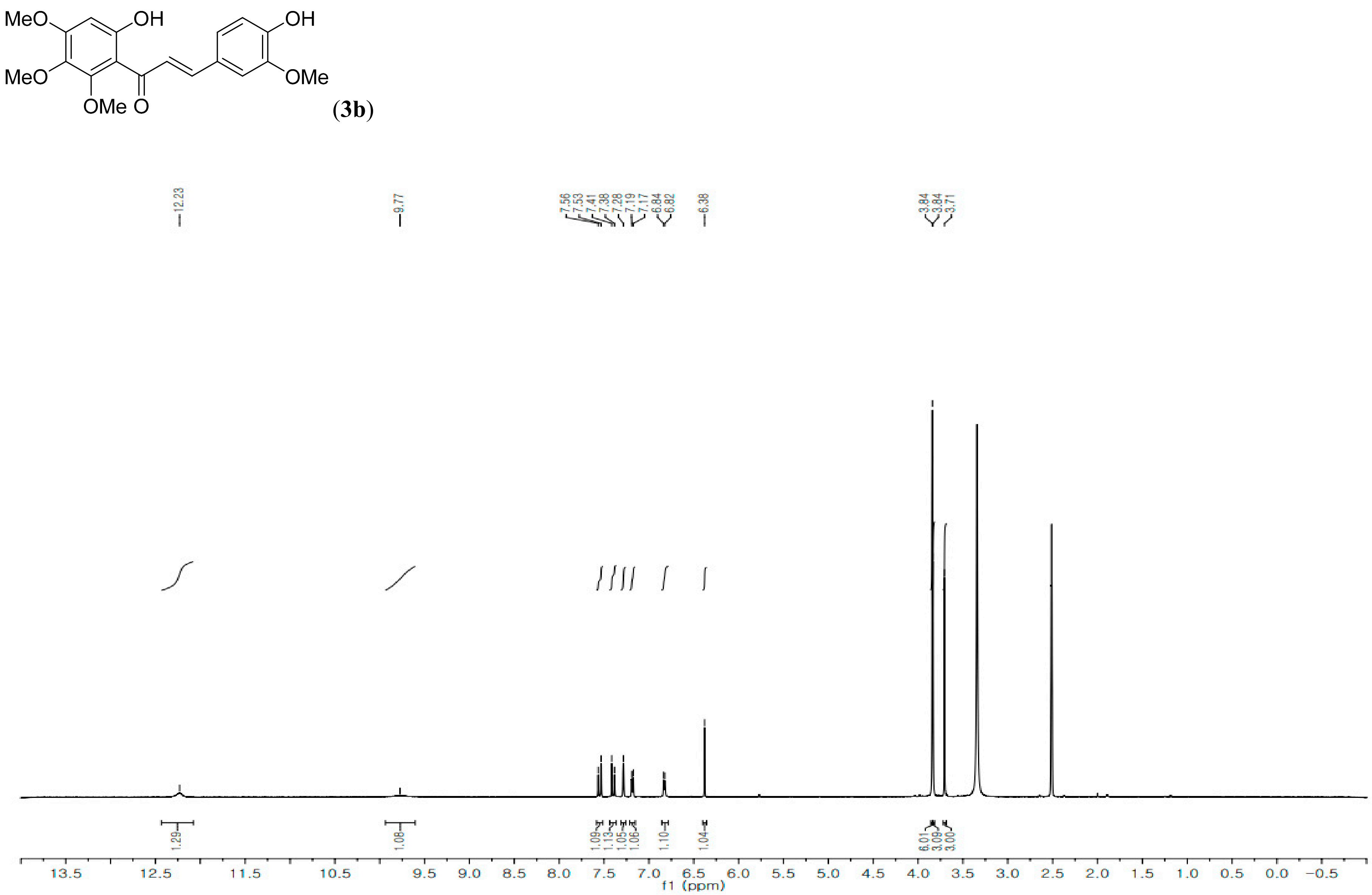
MeO
OH
OH
MeO
OMe
OMe O
(3b)
12.23
9.77
7.56
7.53
7.41
7.38
7.28
7.19
7.17
6.84
6.82
6.38
3.84
3.84
3.71
1.29
1.08
1.09
1.13
1.05
1.06
1.10
1.04
6.01
3.09
3.00
13.5
12.5
11.5
10.5
9.5
9.0
8.5
8.0
7.5
7.0
6.5
6.0
5.5
5.0
4.5
4.0
3.5
3.0
2.5
2.0
1.5
1.0
0.5
0.0
-0.5
f1 (ppm)

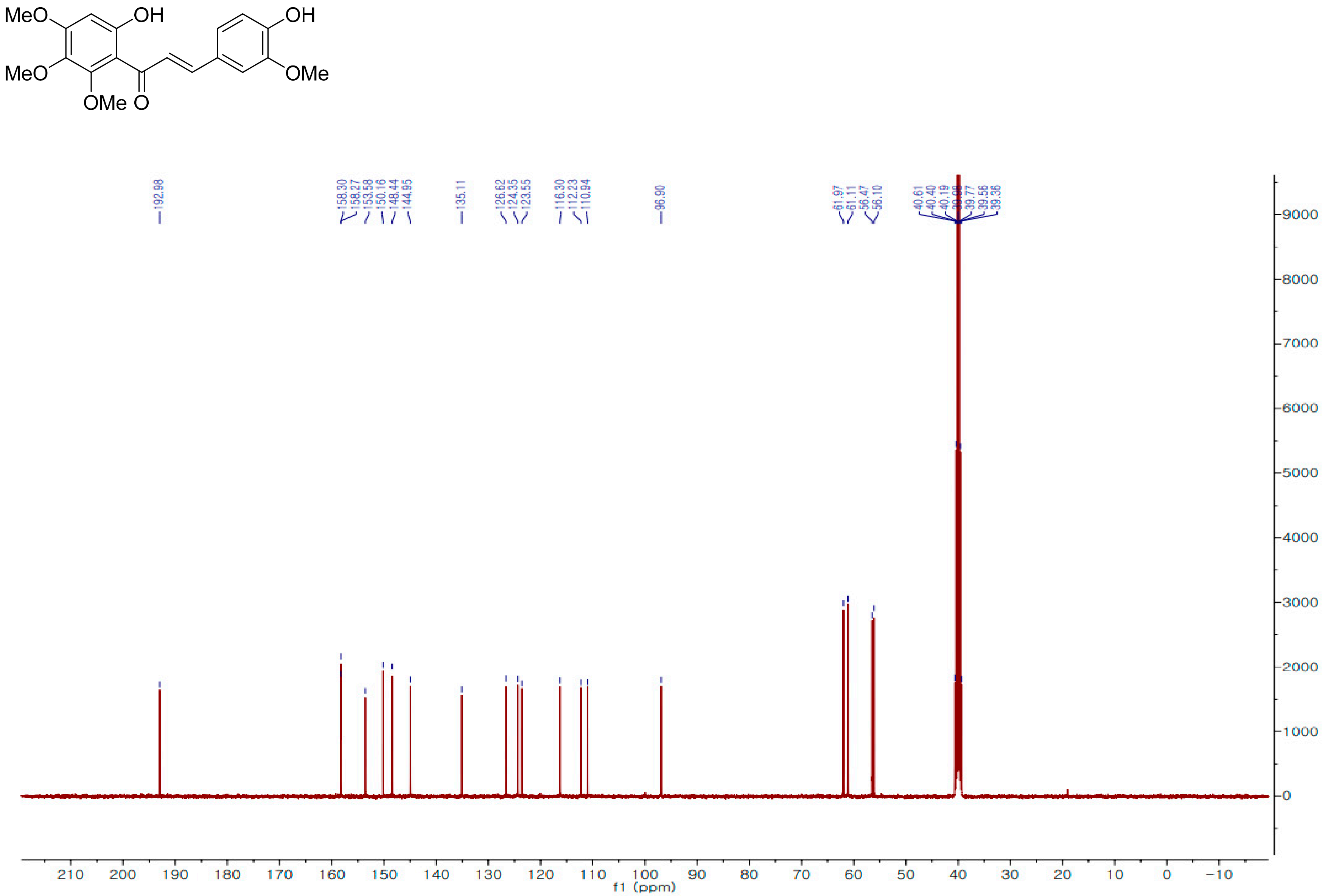
MeO
OH
MeO
OMe
O
OH
OMe
192.98
158.30
158.27
153.58
150.16
148.44
144.95
135.11
126.62
124.35
123.55
116.30
112.23
110.94
96.90
61.97
61.11
56.47
56.10
40.61
40.40
40.19
39.98
39.77
39.56
39.36
9000
8000
7000
6000
5000
4000
3000
2000
1000
0
210 200 190 180 170 160 150 140 130 120 110 100 90 80 70 60 50 40 30 20 10 0 -10
f1 (ppm)

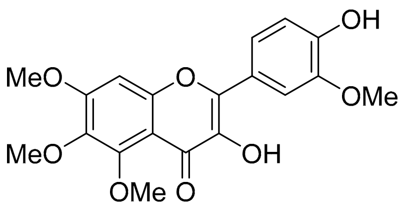
OH
MeO
O
OMe
MeO
OH
OMe O

(4)

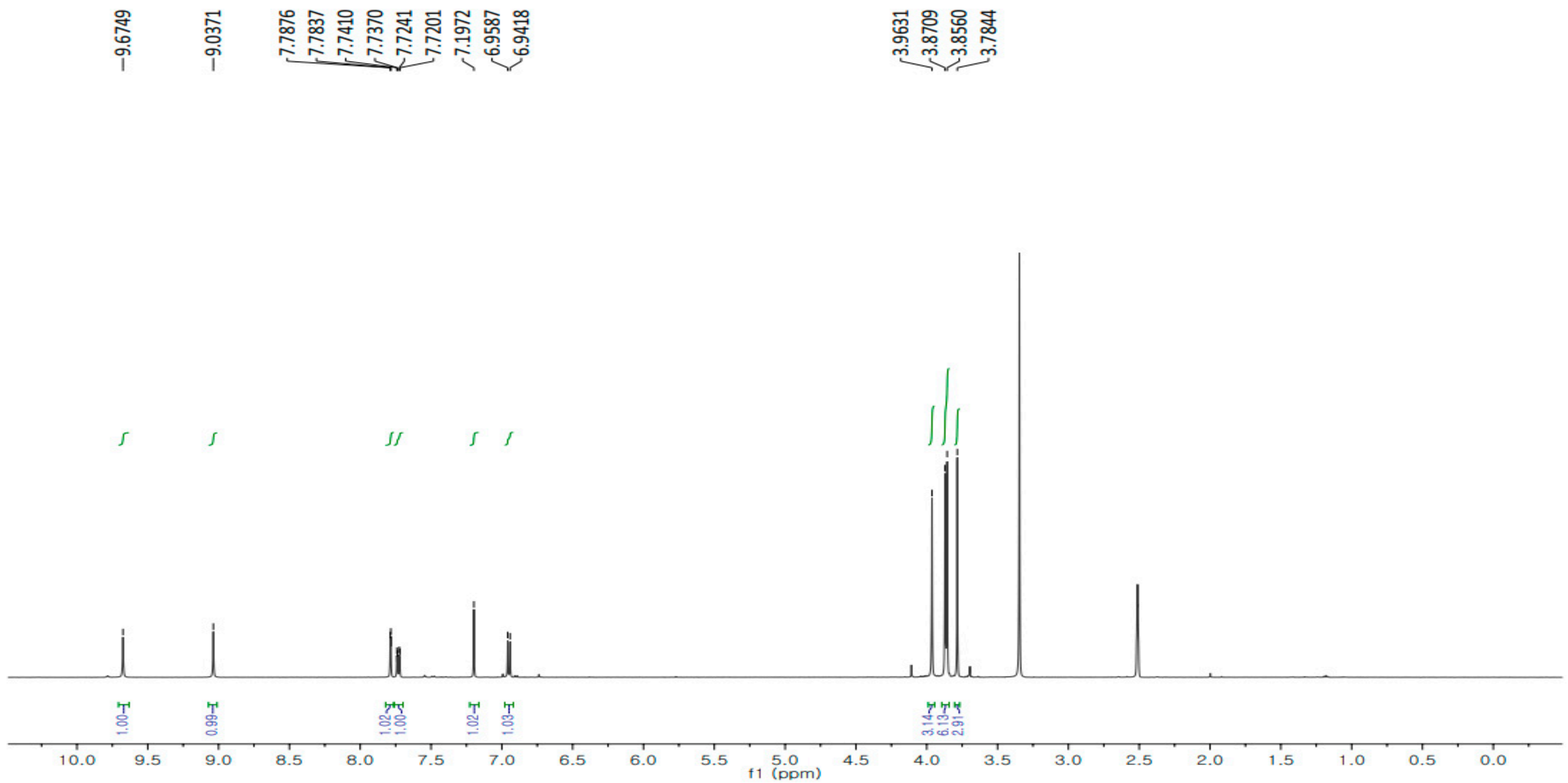
9.6749
9.0371
7.7876
7.7837
7.7410
7.7370
7.7241
7.7201
7.1972
6.9587
6.9418
3.9631
3.8709
3.8560
3.7844
1.00
0.99
1.02
1.00
1.02
1.03
3.14
6.13
2.91
10.0
9.5
9.0
8.5
8.0
7.5
7.0
6.5
6.0
5.5
5.0
4.5
4.0
3.5
3.0
2.5
2.0
1.5
1.0
0.5
0.0
f1 (ppm)

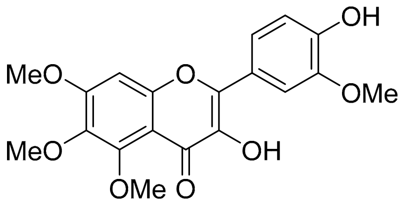
MeO
MeO
OMe
O
O
OH
OH
OMe

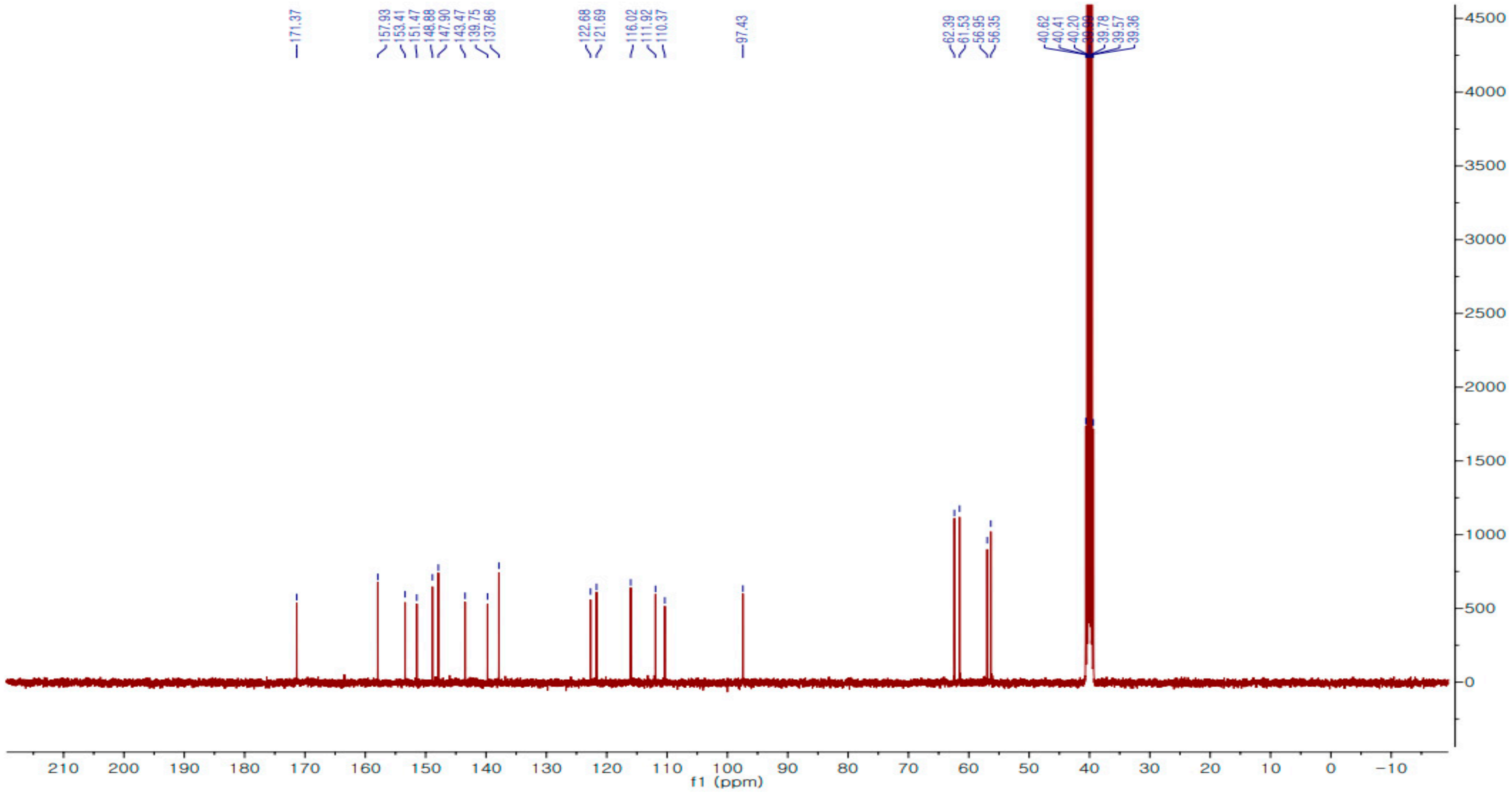
171.37
157.93
153.41
151.47
148.88
147.90
143.47
139.75
137.86
122.68
121.69
116.02
111.92
110.37
97.43
62.39
61.53
56.95
56.35
40.62
40.41
40.20
39.78
39.57
39.36
4500
4000
3500
3000
2500
2000
1500
1000
500
0
210
200
190
180
170
160
150
140
130
120
110
100
90
80
70
60
50
40
30
20
10
0
−10
f1 (ppm)

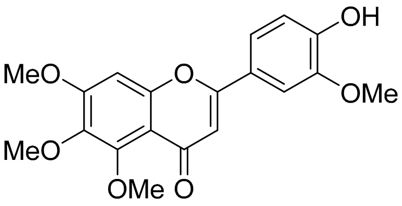

(**5**)

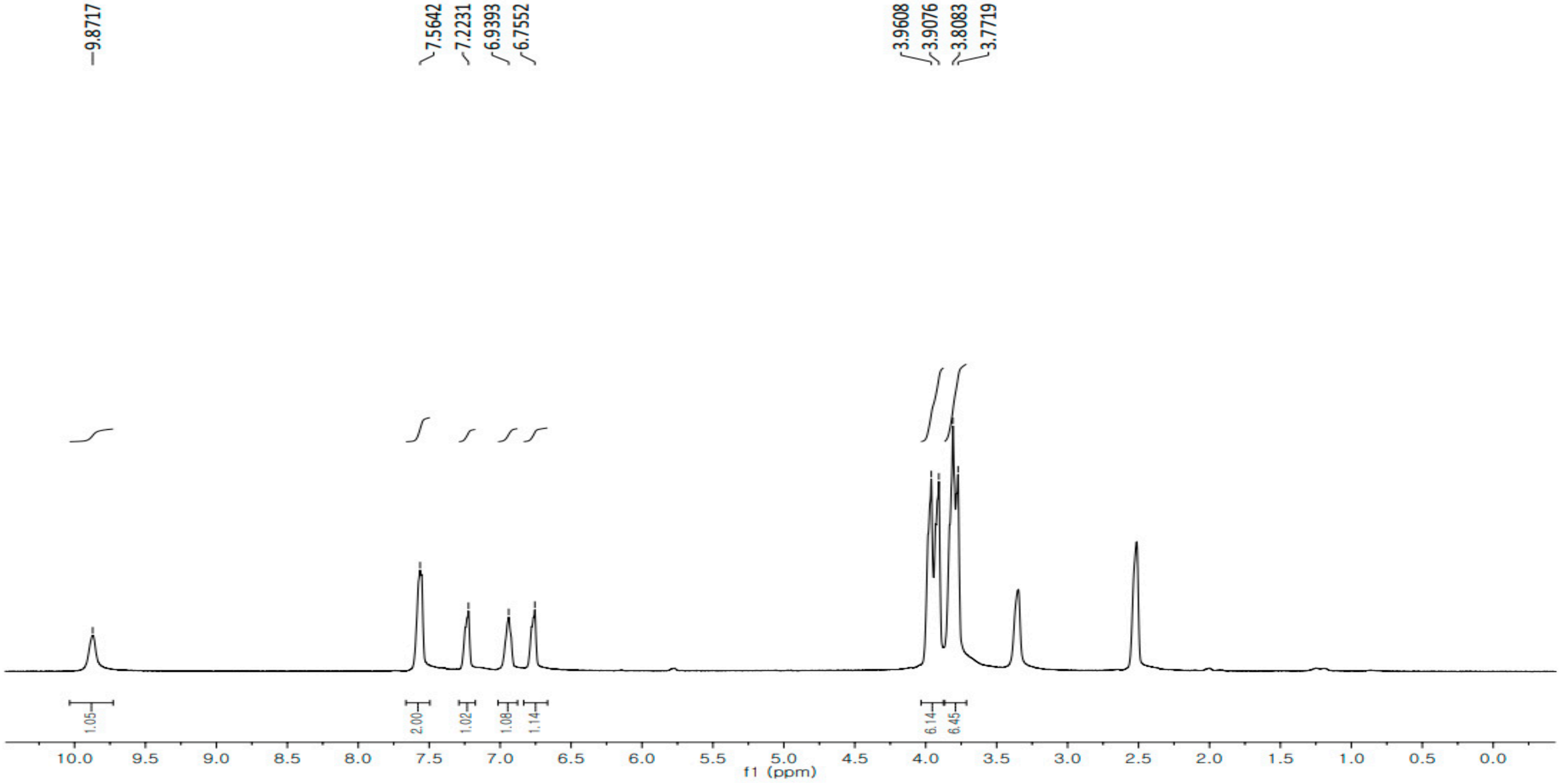

9.8717
7.5642
7.2231
6.9393
6.7552
3.9608
3.9076
3.8083
3.7719
1.05
2.00
1.02
1.08
1.14
6.14
6.45
10.0 9.5 9.0 8.5 8.0 7.5 7.0 6.5 6.0 5.5 5.0 4.5 4.0 3.5 3.0 2.5 2.0 1.5 1.0 0.5 0.0
f1 (ppm)

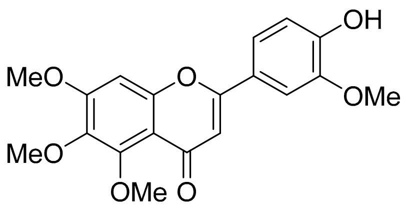

OH
MeO
OMe
MeO
OMe
O

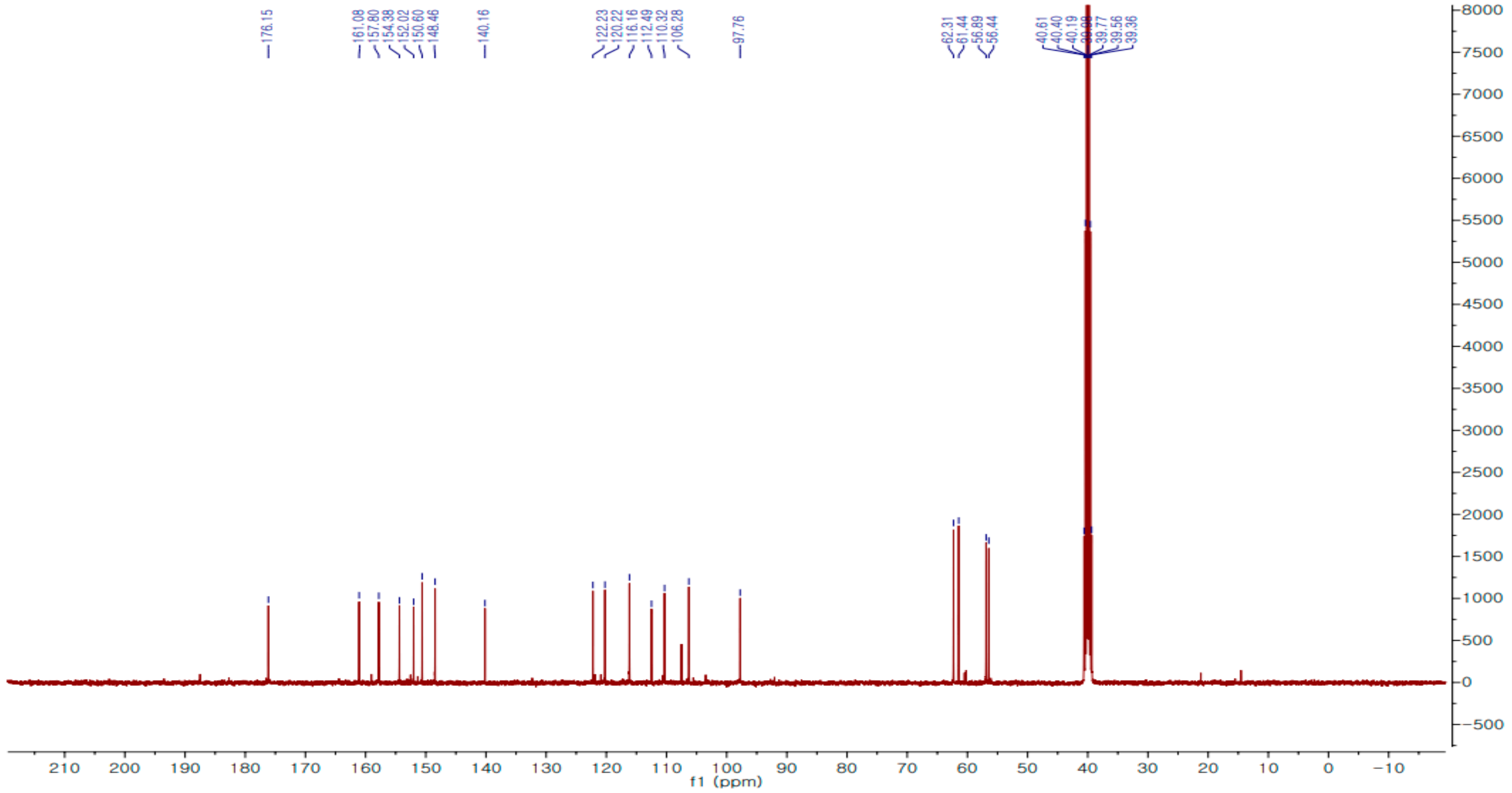

176.15
161.08
157.80
154.38
152.02
150.60
148.46
140.16
122.23
120.22
116.16
112.49
110.32
106.28
97.76
62.31
61.44
56.89
56.44
40.61
40.40
40.19
39.77
39.56
39.36
f1 (ppm)

Crude NMR during demethylation of 5 (after 32 h)

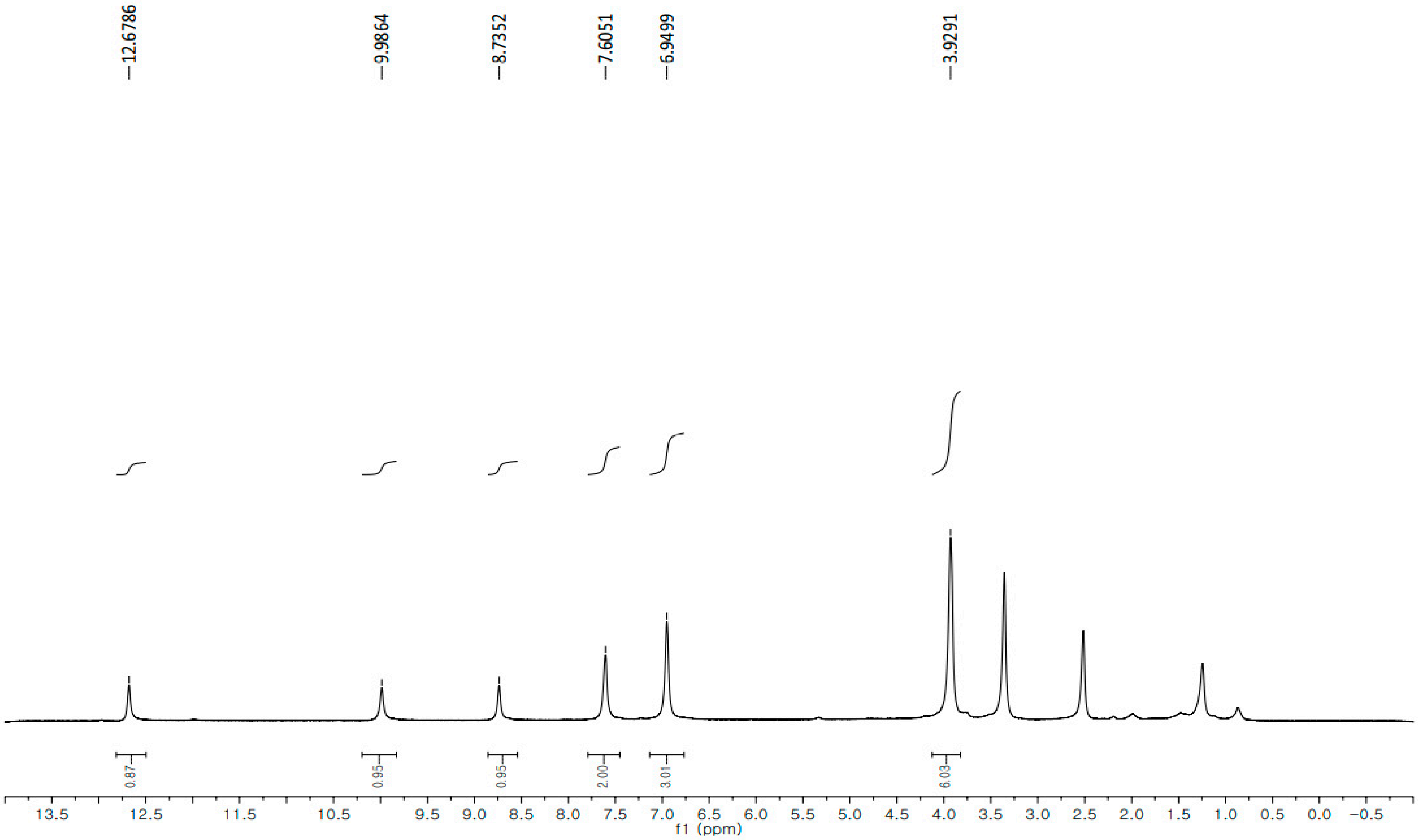

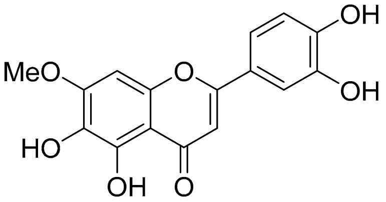
MeO
O
OH
OH
HO
OH
O

(**1**)

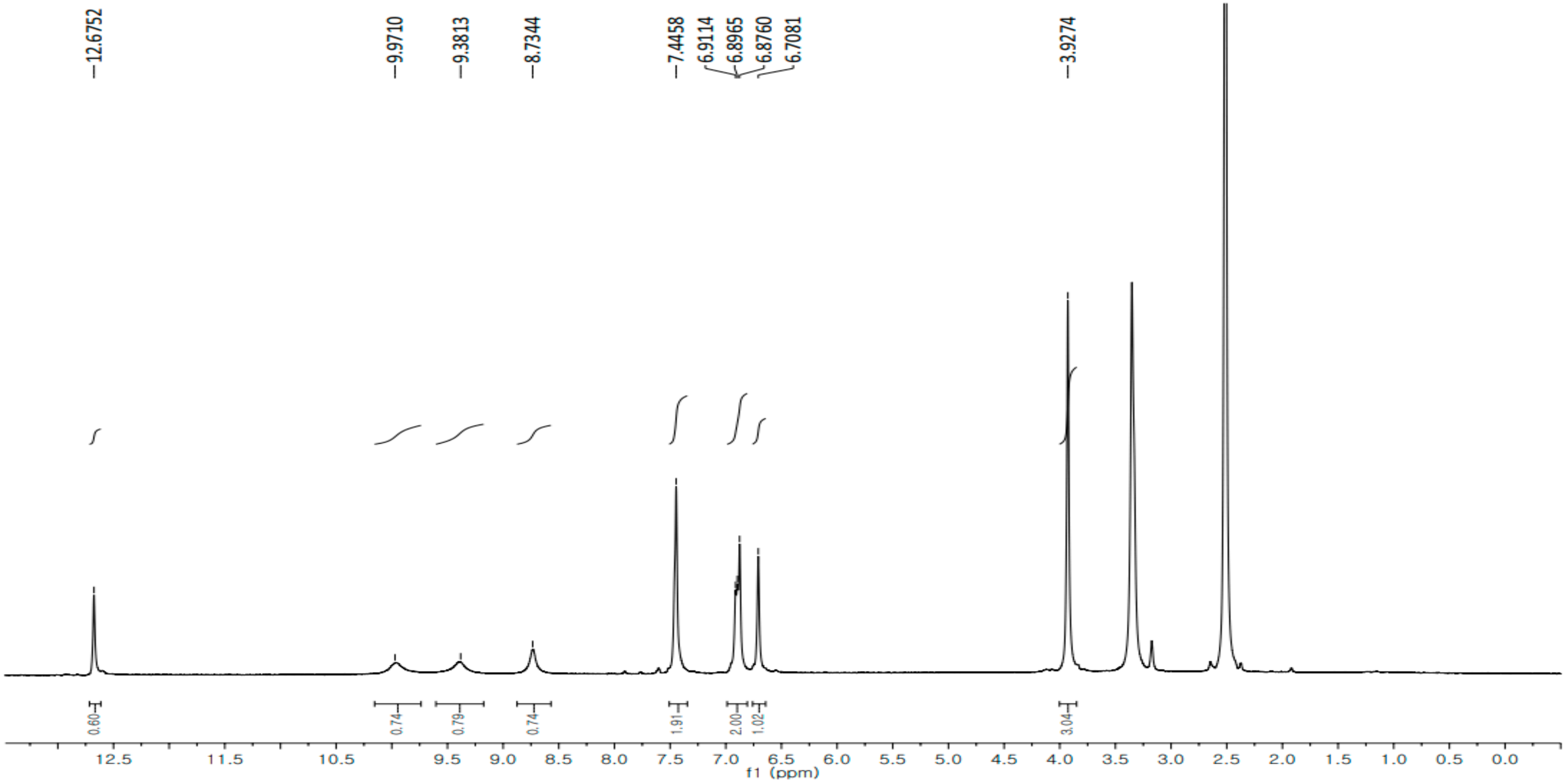
12.6752
9.9710
9.3813
8.7344
7.4458
6.9114
6.8965
6.8760
6.7081
3.9274
0.60
0.74
0.79
0.74
1.91
2.00
1.02
3.04
12.5
11.5
10.5
9.5
9.0
8.5
8.0
7.5
7.0
6.5
6.0
5.5
5.0
4.5
4.0
3.5
3.0
2.5
2.0
1.5
1.0
0.5
0.0
f1 (ppm)

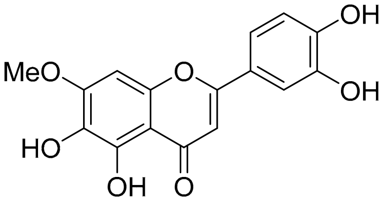
MeO
HO
OH
O
O
OH
OH

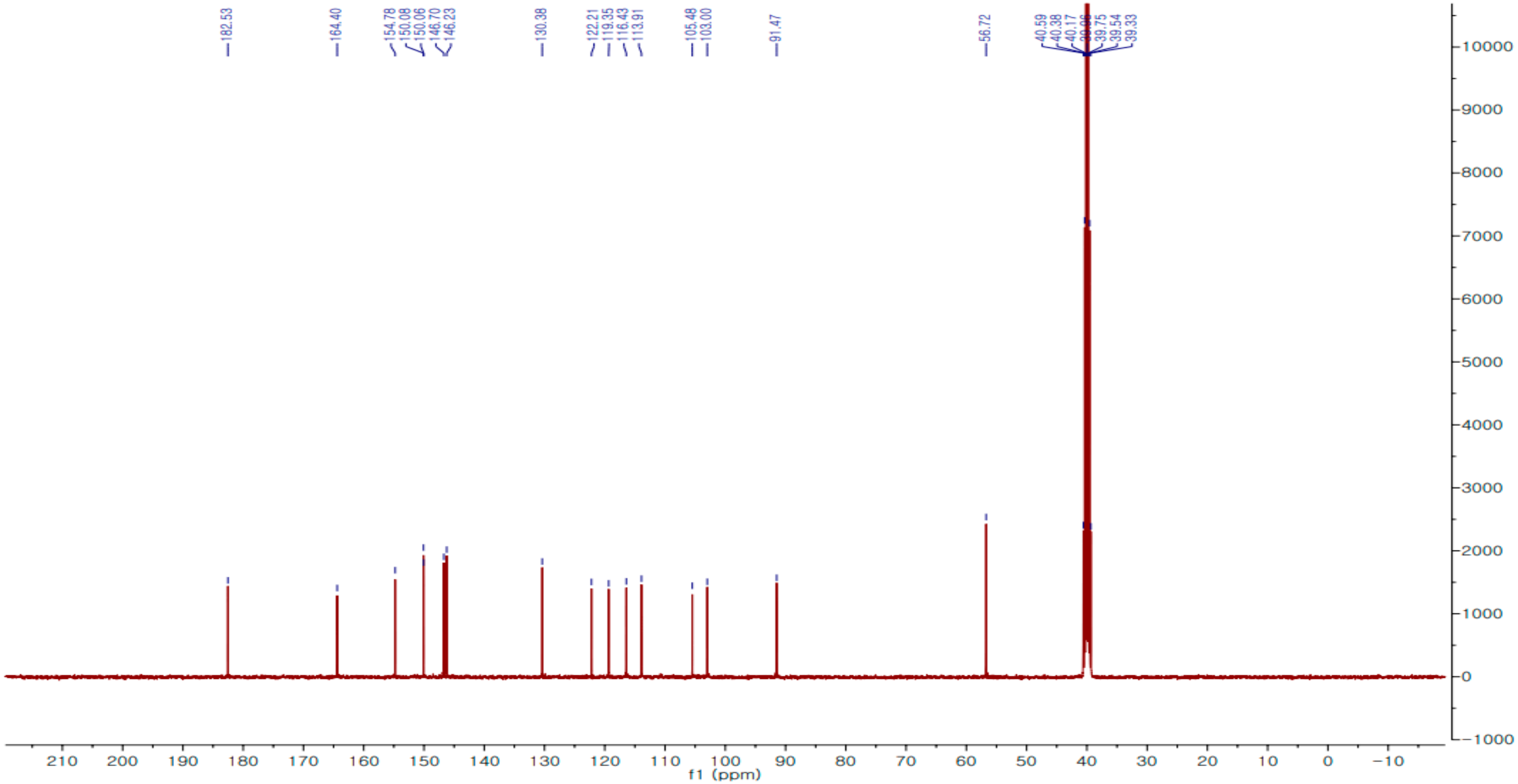
182.53
164.40
154.78
150.08
150.06
146.70
146.23
130.38
122.21
119.35
116.43
113.91
105.48
103.00
91.47
56.72
40.59
40.38
40.17
39.75
39.54
39.33
f1 (ppm)

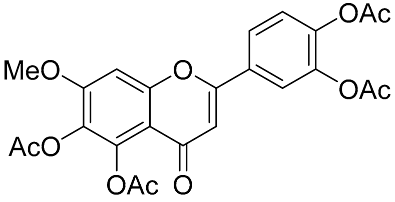
OAc
MeO
O
OAc
AcO
OAc
O

(6)

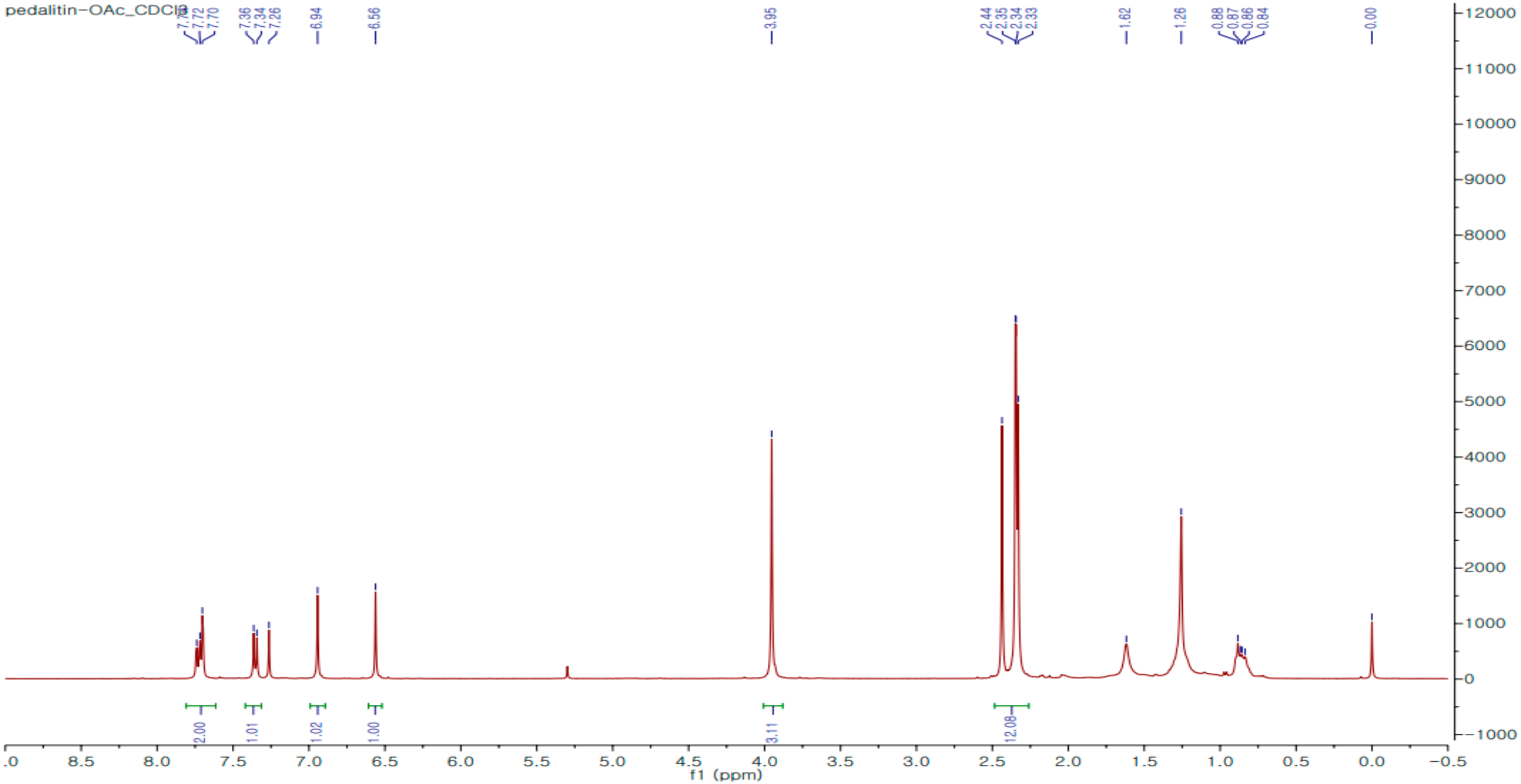
pedalitin−OAc_CDCl3
7.72
7.70
7.36
7.34
7.26
6.94
6.56
3.95
2.44
2.35
2.34
2.33
1.62
1.26
0.88
0.87
0.86
0.84
0.00
2.00
1.01
1.02
1.00
3.11
12.08
f1 (ppm)

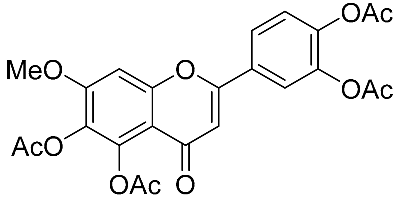

MeO
O
OAc
OAc
AcO
OAc
O

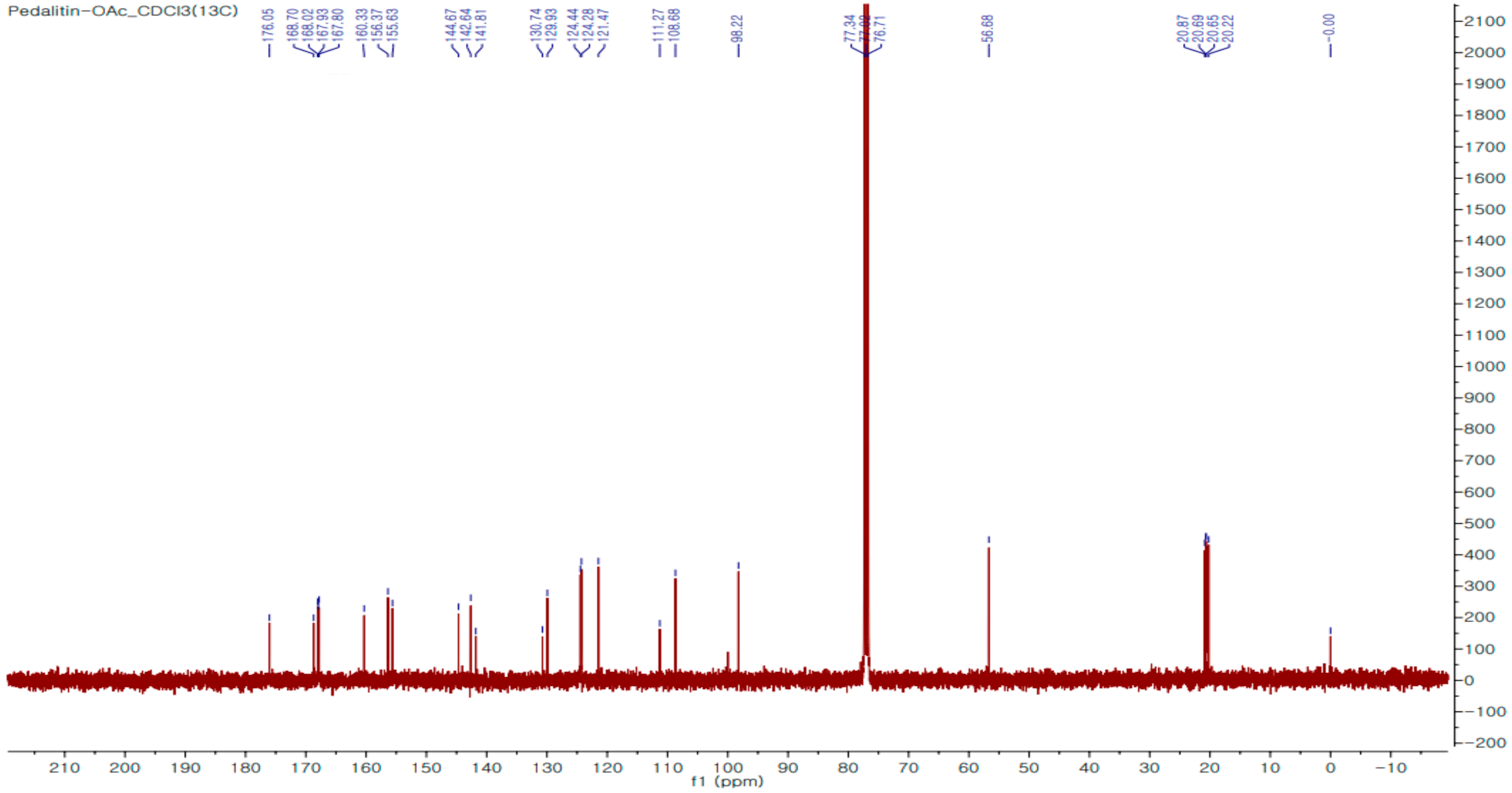

Pedalitin-OAc_CDCl3(13C)
176.05
168.70
168.02
167.93
167.80
160.33
156.37
155.63
144.67
142.64
141.81
130.74
129.93
124.44
124.28
121.47
111.27
108.68
98.22
77.34
76.71
56.68
20.87
20.69
20.65
20.22
-0.00
f1 (ppm)
